# Nano-architecture of gustatory chemosensory bristles and trachea in *Drosophila* wings

**DOI:** 10.1038/srep14198

**Published:** 2015-09-18

**Authors:** Jean Christophe Valmalette, Hussein Raad, Nan Qiu, Satoshi Ohara, Maria Capovilla, Alain Robichon

**Affiliations:** 1 Aix Marseille Université, CNRS, IM2NP, UMR 7334, Case 907, 13288 Marseille, France; 2 Université de Toulon, CNRS, IM2NP, UMR 7334, 83957 La Garde, France; 3 UMR INRA/CNRS/UNS 1355/7254, Institut Sophia Agrobiotech, 400 route des Chappes, P. O. Box 167, 06903 Sophia Antipolis, France; 4 JWRI, Osaka University, 11-1, Mihogaoka, Ibaraki, Osaka 567-0047, Japan

## Abstract

In the *Drosophila* wing anterior margin, the dendrites of gustatory neurons occupy the interior of thin and long bristles that present tiny pores at their extremities. Many attempts to measure ligand-evoked currents in insect wing gustatory neurons have been unsuccessful for technical reasons. The functions of this gustatory activity therefore remain elusive and controversial. To advance our knowledge on this understudied tissue, we investigated the architecture of the wing chemosensory bristles and wing trachea using Raman spectroscopy and fluorescence microscopy. We hypothesized that the wing gustatory hair, an open-ended capillary tube, and the wing trachea constitute biological systems similar to nano-porous materials. We present evidence that argues in favour of the existence of a layer or a bubble of air beneath the pore inside the gustatory hair. We demonstrate that these hollow hairs and wing tracheal tubes fulfil conditions for which the physics of fluids applied to open-ended capillaries and porous materials are relevant. We also document that the wing gustatory hair and tracheal architectures are capable of trapping volatile molecules from the environment, which might increase the efficiency of their spatial detection by way of wing vibrations or during flight.

Insect wings are an evagination of the exoskeleton and their membrane is formed by two closely apposed layers of integument^1^,^2^,^3^. The two integument layers remain separate in the veins, where they usually thicken and sclerotize^1^,^2^,^3^. The veins create a network of pipes that shelter air-filled tracheal tubes surrounded by a haemolymph layer^1^,^2^,^3^. The haemolymph flows into the wing veins as an extension of the body haemocoel^1^,^2^,^3^. In parallel, the environmental air diffuses into the wing tracheal system *via* the body tracheal pipe network^3^. Importantly, a tracheal tube and a nerve coexist within the dorsal anterior margin vein where the wing mechano- and gustatory sensilla are closely associated^4^,^5^,^6^,^7^,^8^,^9^. This generates a sophisticated exchange structure between the haemocoel, air and living cells of the sensilla. The evolutionary success of the insect wing neuroanatomy results in a sophisticated flight guidance partly by providing volatile and hydrophilic compounds to the sensilla cells^1^,^2^,^3^.

Two types of bristles exist along the anterior *Drosophila* wing margin vein^7^,^10^. The short and robust bristles are part of the mechanoreceptor sensilla, whose main role consists of sensing mechanical information such as touch, vibration and air turbulence. These mechanical bristles alternate with long and thin hairs that shelter the dendrites of the gustatory neurons for which the functions and transduction pathways are poorly documented^7^,^8^,^10^,^11^,^12^. In *Drosophila*, the wing gustatory chemosensory sensilla are each composed of 2–4 neurons and one mechanosensory neuron^7^,^8^. Three accessory sheath cells surround these neurons: tormogen, trichogen and thecogen cells^10^,^11^,^12^. Surprisingly, in the anterior wing margin, the chemosensory sensilla harbour gustatory instead of olfactory receptors.

Until now, little has been reported regarding the physiology of neurosensory cells in the insect wing. Most electrophysiology studies failed to provide consistent and repeatable results in this tissue, although *a priori* conditions seem ideal in terms of the accessibility of external ligands to the receptors through the pore at the extremity of the chemosensory hair. Many studies using powerful genetic tools in the *Drosophila* model^11^,^12^ along with ultrastructural analysis using scanning electron microscopy^4^,^7^ have abundantly documented the insect wing neuroanatomy and morphology. In contrast, the complex physiology of the insect wing remains understudied. One explanation might be that the chemosensory neurons of wings should be protected against the mechanical stress and heat generated by wing vibrations during flight^13^,^14^. This does not apply to the neurons that are sheltered in the contractile proboscis or in the legs, for which many electrophysiology data have been reported^12^. The wing vibrations during flight would likely expel the lymph through the pore and dry the dendrites if evolution had not engineered a structural architecture to prevent this. In other words, if we assume that the vibrational component of the insect wing is part of the process of wing chemodetection, the air vortex created by the flapping wings is likely required to facilitate the accessibility of environmental compounds to the wing neuronal receptors. In such a case, a partially air-filled bristle would be a more efficient system to trap environmental molecules such as volatile compounds and/or hydrophilic molecules solubilized in water microdroplets than a hair filled with lymph up to its extremity.

The absorption/desorption of gas or liquids and the transition vapour to liquid in a porous material is a well-documented phenomenon^15^,^16^,^17^,^18^,^19^,^20^,^21^ that should apply to open-ended insect hairs along with tracheal pipes. Water vapour condenses in a liquid state inside open carbon nanotubes^15^,^16^,^17^,^18^,^19^,^20^,^21^. An increased and high number of van der Waals interactions among water molecules occurring inside the confined space of a capillary provoke the vapour to liquid transition, and this occurs when the vapour density in the atmosphere is lower than the saturation point^15^,^16^,^17^,^18^,^19^,^20^,^21^. This phenomenon leads to molecule trapping in a confined space of porous material, which cannot be achieved on smooth surfaces^15^,^16^,^17^,^18^,^19^,^20^,^21^. The vapour to liquid phase transition in confined geometries has many applications in chemical sensing, although the basic mechanisms of capillary condensation and adsorption are still poorly understood^22^,^23^,^24^,^25^. We hypothesize that an insect sensorial hair made of chitin with an apical pore and hollow inside (even though it is partially filled with lymph) corresponds to the physical attributes of an empty, open capillary. In the same way, the insect tracheal system made up of a network of microtubes is similar to a porous material obeying to the same physical principles.

Here, we investigated the process through which volatile molecules found in nature such as ethanol, organic acids (propionic and acetic acids) or hydrophilic molecules dissolved in water microdroplets enter and condense inside the chemosensory hair space of the *Drosophila* wing. This should also apply to the overall network of air-filled tubes that constitutes the tracheal system. The first goal of this study is therefore to probe the content of the hollow hairs that shelter the taste receptors in the *Drosophila* wing. We used Raman spectroscopy methods for two reasons. First, the fingerprints of vibrational frequencies are well known and are characteristic of chemical bonds or molecules^26^,^27^,^28^,^29^. Second, these fingerprints can be found directly in complex biological tissues without destructive extraction procedures^26^,^27^,^28^,^29^. Raman spectroscopy is a powerful tool to identify molecules or chemical groups by analysing their vibrational modes^26^,^27^,^28^,^29^. The lateral resolution of Raman spectroscopy imaging (approximately 0.5 micron) provides not only the identification of compounds but also their spatial distribution below the dimension of the hair diameter^26^,^27^,^28^,^29^. We analysed the *Drosophila* wing gustatory hairs by tracking the vibrational mode(s) of molecule(s) associated with lymph compounds. For example, the amide I and II groups of proteins and also aromatic amino acids such tryptophan, phenylalanine and tyrosine show characteristic Raman modes in the 1,200–2,000 cm^−1^ region^26^,^27^,^28^,^29^. We investigated this spectral region to establish whether the interior beneath the pore is empty or filled with lymph. We reasoned that Raman analysis using a focused laser beam with a diameter below 0.5 μm, smaller than the hairs, could be used to probe whether the air/lymph meniscus is close to the pore or inside the hair. The penetration depth of the light probe, sensitivity, photo-degradation, emitted fluorescence and to a lesser extent spatial resolution strongly depend on the excitation wavelength^26^,^27^,^28^,^29^. For these reasons, we used a green line of an argon laser at 514 nm and a near infrared diode at 785 nm. Raman spectral imaging is performed directly on intact chemosensory hairs through the cuticular barrier, which allows us to qualify and quantitate the signatures of chemical bonds as amide and amino acid residues in lymph proteins at the different levels of the hairs. The envelope of these hairs is composed of a rigid matrix of polysaccharides (chitin)^30^,^31^,^32^, which provides the advantages of minimizing the interferences with proteins in Raman spectrometry analysis and greatly improving the signal-to-noise ratio. In addition, we investigated whether the wing tracheal system condenses volatile molecules present in the environment. To investigate this, we used benzaldehyde, which has three properties. It is volatile and aromatic, its aldehyde group forms covalent bonds with the lysine or amine groups of proteins and its benzyl group auto fluoresces when attached to proteins. The increased autofluorescence generated in the wing was analysed as an index of the benzaldehyde accumulation in the trachea and sensory hairs.

The wing beat produces air turbulence called the “leading edge vortex” that spirals off along the anterior wing margin, precisely where the chemo- and mechanosensory hairs are located^13^,^14^. All these elements (air vortex and capillary fluid dynamics) fit a model for which the insect wing chemosensory hairs along with the wing tracheal pipes are structurally designed for an optimal efficiency of chemical detection. Our data support the hypothesis that the wing gustatory hairs are filled with air beneath the pore and that the wing trachea condenses environmental organic volatile molecules. Together, these elements suggest that the wing gustatory sensilla might obtain environmental information from both the tracheal pipes and the hollow hairs with an opened pore at their extremity.

## Results

### Morphological structure of the gustatory sensilla along the anterior margin nerve of the *Drosophila* wing

**Figure 1:**
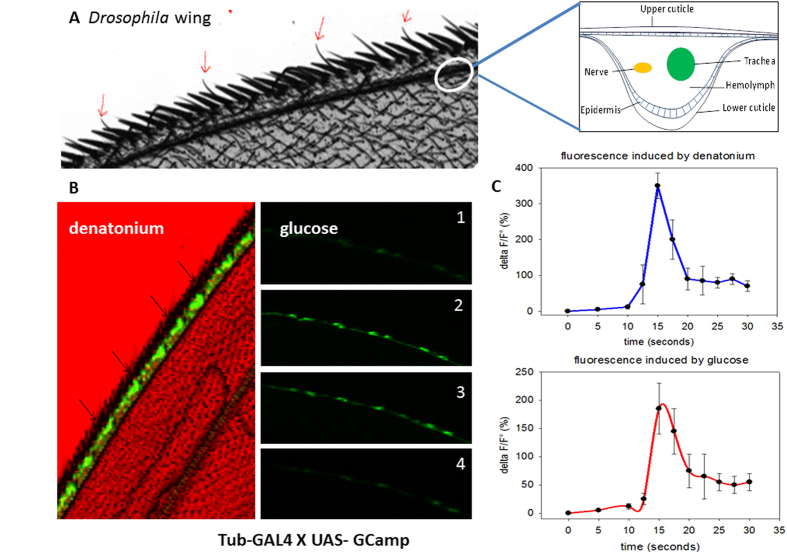
Distribution of gustatory sensilla and calcium imaging in *Drosophila* wings. (**A**) The photograph shows a wing subset corresponding to the anterior wing margin. The stout bristles are mechanosensilla and the thin hairs (red arrows) are chemosensilla that shelter gustatory neurons. The dark line along the anterior margin is the vein of the anterior wing margin that contains haemolymph, trachea and the nerve made of bundles of axons of the mechanosensory and chemosensory (gustatory) neurons (see the schematic of a cross section in the box at the right). (**B**) The photographs display calcium imaging by a fluorescent probe. The gustatory sensilla light up after denatonium (1 mM) or glucose (50 mM) binding to their respective receptors. The wing corresponds to the F1 progeny of a cross between homozygous *UAS-GCamp* and heterozygous *Tub-GAL4* flies. The photographs (1 to 4) represent successive sequences over a total period of 20 seconds after glucose stimulation (1 is the time 0 before induction). (**C**) Time course of the fluorescence after denatonium (1 mM) or glucose (50 mM) stimulation. The graphs represent the average of the determination (delta F/F0) for 10 wings and 3 sensilla per wing (n = 30, mean + /−SE, p < 0.0005 between the 15 and 30 sec., Student’s t-test).

**Figure 2:**
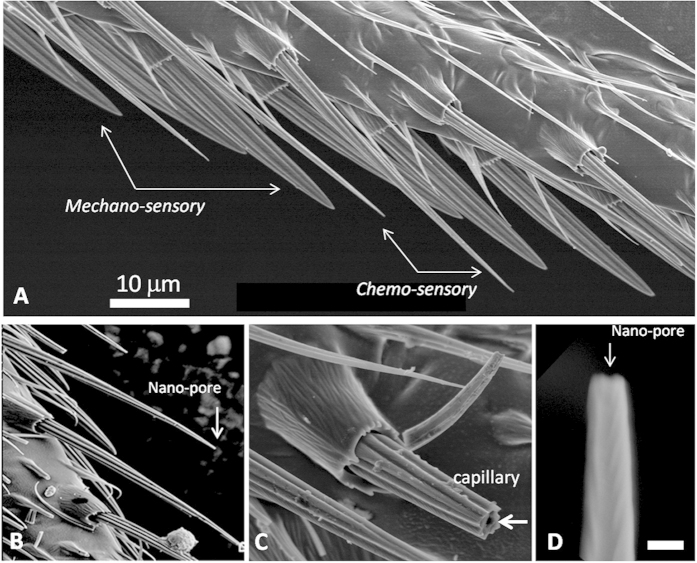
Electron microscopy photographs of the *Drosophila* wing bristles of the anterior margin. (**A**) The wing anterior margin has two types of bristles. The stout and short ones are linked to the mechanosensilla, while the long hairs are associated with the gustatory sensilla. (**B**) Sensorial bristle made of chitin with a nano-pore at the top. (**C**) An opening made with a razor shows that the thin hairs are hollow tubes. These interior spaces shelter the lymph and the dendrites of sensilla neurons. (**D**) Collapsed nano-pore located at the apex of the bristle (scale bar = 300 nm).

The photograph in Fig. 1A shows the border of the anterior wing margin where the stout bristles alternate with thin and long hairs (thin red arrows). The former are associated with the mechanoreceptor sensilla, while the latter host gustatory sensilla. These thin hairs are hollow tubes opened to the environment by a unique pore at their extremity^7^. The photographs in Fig. 1B show the fluorescence emitted by a calcium sensor (G-Camp), which is a hybrid of a modified GFP linked to a calcium binding motif that emits fluorescence upon Ca^2+^ binding^33^. We observed that the bitter molecule denatonium and the sweet molecule glucose produced significant signals in the sensilla. Consecutive snapshots (1 to 5) over a period of twenty seconds after glucose stimulation are shown (Fig. 1B). These flashes oscillated during the time course of activation. A time course of emitted fluorescence (ΔF/F°) is presented on a large number of sensilla recruited in a panel of 10 individual flies. This assay and the genetic construct based on a ubiquitous strong promoter (*tub-Gal4*) to drive the calcium sensor allowed us to unambiguously demonstrate the functionality of taste receptors in *Drosophila* wings. Figure 2 illustrates the morphology of these bristles revealed by electron microscopy. Damage caused by a razor (arrows) highlights that the interior is empty, which confirms that these bristles are hollow tubes.

### Probing the hollow thin hair by Raman spectroscopy

**Figure 3:**
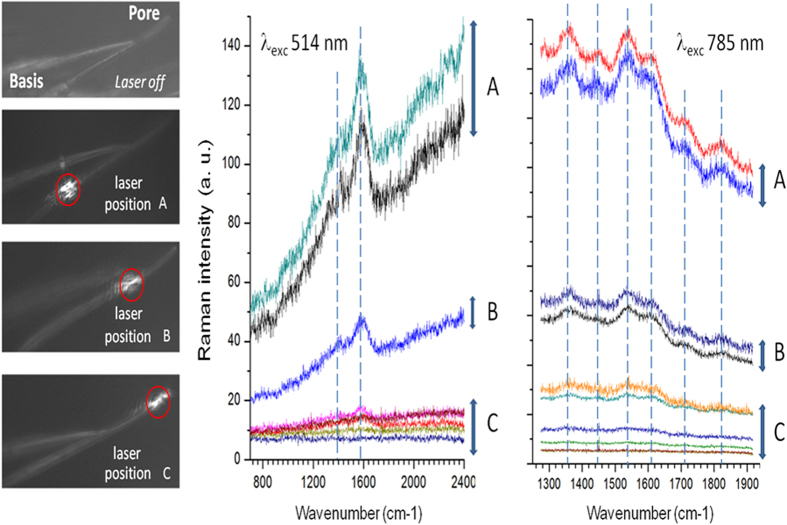
Raman spectra of *Drosophila* chemosensory wing hairs under 514 nm and 785 nm laser excitation. Each chemosensory wing hair shows a diameter little changed from the base to the extremity, where a unique pore is located. The Raman beam was focused on three places: at the base of the bristle (**A**), at 2/3^rd^ of the length (**B**) and at 9/10^th^ of the length (near the extremity) (**C**). On the left, microphotographs represent one hair of a wing with the impact of the laser beam. Excitation lengths were 514 nm (middle) and 785 nm (right). The three corresponding spectra are represented on each graph. We noted a large main peak between 1,500 and 1,650 cm^−1^. Each individual line in the three categories (**A**–**C**) was obtained with different individual wings. Strong differences in the intensity of Raman signals are shown among the three locations of the laser beam.

The anterior wing margin contains a trachea (an air-filled tube) and a nerve both bathed by a haemolymph layer to feed the sensilla cells (box in Fig. 1A). However, the opened thin hairs shelter dendrites that are bathed with lymph secreted by the apical side of sensilla cells, but are separated from the haemolymph vein^7^. The comparative Raman spectra along the hair from the base to the extremity are shown in Fig. 3 for two excitation wavelengths (514 and 785 nm). Under 514 nm excitation (Fig. 3, middle), the diameter of the probed volume was approximately 0.28 μm, which is smaller than the hair diameter. The spectra showed a strong fluorescence background and two broad bands at approximately 1,360 and 1,580 cm^−1^. These bands, respectively assigned in the literature to the D-band and G-band of carbon compounds^26^,^27^,^28^,^29^, are explained by the carbon residues resulting from the thermal breakdown of proteins induced by the laser beam, even under a very low power density (<12 μW). However, the Raman spectra varied along the hair, with low responses near the tip (Fig. 3, position C), suggesting a strong position-dependence of the intensity of the signals.

**Figure 4:**
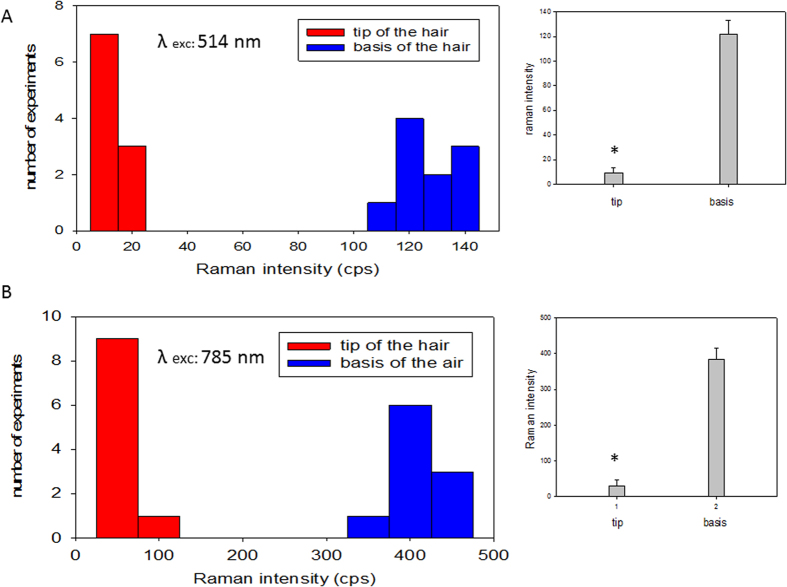
Statistical analysis of the Raman spectra under 514 nm and 785 nm laser excitation. Ten determinations each on the tip and the base of arbitrary gustatory wing hairs were analysed for each excitation wavelength. Each of these measures was performed on different wings. The statistical analysis was carried out using Student’s t-test and is represented at the right (bars are the mean + /−SE, n = 10, *p value < 0.0005 between the measurements at the tip and the base of the hairs).

To avoid the fluorescence induced by the excitation wavelength and the degradation of organic molecules by the laser beam, we performed Raman experiments under the 785 nm excitation (Fig. 3, right). As a consequence, this led to a larger probed volume and a lower spatial resolution (approximately 0.45 μm) compared to those obtained under the 514 nm excitation. The spectra showed several vibrational features. A mode at 1,540 cm^−1^ corresponds to the position of the amide II bands resulting from the combination of N-H bending and C-N stretching. Another mode at 1,610 cm^−1^ could be assigned to the C=O stretching of amide I. Finally, the 1,350 cm^−1^ band fits with the position of C-N stretching and N-H bending of aromatic amino acids, which is consistent with the expected presence of tryptophan, phenylalanine and tyrosine. As previously observed under 514 nm excitation, we obtained strong differences in Raman intensity depending on the laser beam position along the hair, with a loss of signal close to the pore (Fig. 3, position C). Although the Raman vibrational frequencies are sensitive to the local environment, the obtained spectra are “signatures” of molecular motifs such as amide I and II and also aromatic amino acids such as tryptophan, phenylalanine and tyrosine. The band positions revealed the conjugated presence of these molecular motifs and the overlap of the corresponding peaks. For both excitation wavelengths, the Raman intensity along the hair drastically changed when crossing a “frontier” position B (Fig. 3). We noticed high and almost constant Raman signals before this frontier (position A) and very low signals after this position towards the apical pore (position C). Considering these data and the fact that the hair diameter appeared unchanged from its base to its extremity, we can state that a lymph meniscus existed approximately one third of the hair length beneath the pore. The frontier position migrated slightly towards the base of the hair after a period of one hour, suggesting that the lymph dried slowly after wing dissection (all Raman spectra reported in Fig. 3 were obtained within a few minutes after dissection). Finally, a statistical analysis was performed on ten determinations at the base or the tip and each measure was performed on one arbitrary gustatory hair. Ten wings were examined for each set (Fig. 4). We observed significant differences in Raman intensity depending on the position of the laser beam. In contrast with high intensity signals at the basis of the hair, very low signals at the tip of the hair are systematically obtained, suggesting an air layer beneath the pore.

### Probing benzaldehyde condensation in the trachea and in the chemosensory hairs of *Drosophila* wings

**Figure 5:**
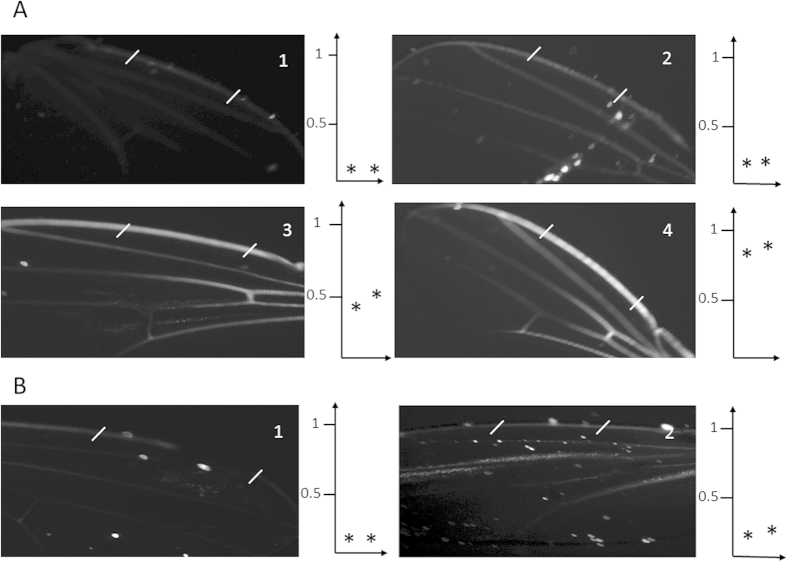
Autofluorescence generated by benzaldehyde in the veins of a *Drosophila* wing. (**A**) The four photographs represent exposure to benzaldehyde (10 μl on a paper towel soaked in water and placed in a 200 ml *Drosophila* vial) of living seven day old *Drosophila* females. 1–4 represent the time of exposure at 0, 1, 2 and 4 hours, respectively. At the right of each photograph is shown the relative intensity of the autofluorescence corresponding to the two white cross traits from a range of 0 to 1. The asterisks correspond to the average of five experiments and represent the mean + /−SE. 0 h: 0.15 + /−0.05 for both stars; 1 h: 0.3 + /− 0.1 for both stars; photo 2 h: 0.45 + /−015 and 0.55 + /−0.25, respectively; 4 h: 0.75 + /− 0.2 and 0.85 + /−0.5, respectively. A Student’s t-test analysis gives a p < 0.001 between the corresponding determinations at 2 and 4 hours (photos 3 and 4). (**B**) The same experiment was conducted in identical conditions, but cut wings were used instead of living animals. The relative fluorescence intensity corresponding to white traits in the photographs are represented in the graphs at the right (average of n = 5 experiments). In cut wings, no significant differences were observed between 1 and 2 that correspond to the time of exposure at 0 and 4 hours, respectively. We observed that the benzaldehyde covalent binding marks the tracheal network in wings, and this labelling requires living animals with an intact respiratory system.

**Figure 6:**
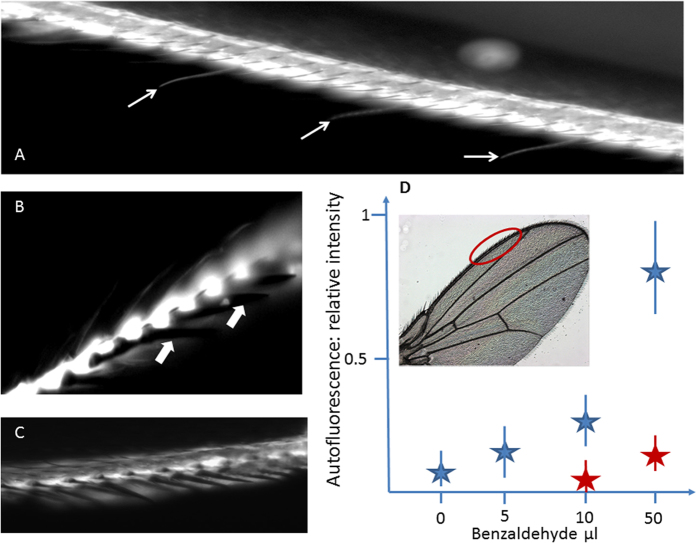
Autofluorescence generated by benzaldehyde in the chemosensory hairs and the anterior margin vein of the *Drosophila* wing. Flies were exposed to a high dose of benzaldehyde (50 μL in a 300 ml vial for two hours). (**A**) The anterior wing vein is fluorescent, and the thin hairs corresponding to the chemosensory sensilla (thin arrows) are also labelled. (**B**) The photograph shows fluorescence in the chemosensory hairs but not in the stout bristles corresponding to the mechanoreceptors (thick arrows). The vein is intensely labelled. (**C**) Fluorescence obtained with a cut wing treated in the same conditions. The margin vein is weakly labelled, but the thin chemosensory hairs are not labelled at all. (**D**) Dose response of emitted fluorescence obtained with living flies (blue stars) after two hours of exposure to increasing concentrations of benzaldehyde. The fluorescence was measured in the anterior wing margin region delimited by the red oval in the box. Each determination is the mean + /− SE, n = 10. A relative measure of fluorescence obtained within the anterior wing margin of cut wings treated in the same conditions (red stars) is reported for two concentrations of benzaldehyde (mean + /−SE, n = 5). For 10 and 50 μl benzaldehyde, p < 0.01 and < 0.001, respectively, between the cut and living wings (Student’s t-test).

**Figure 7:**
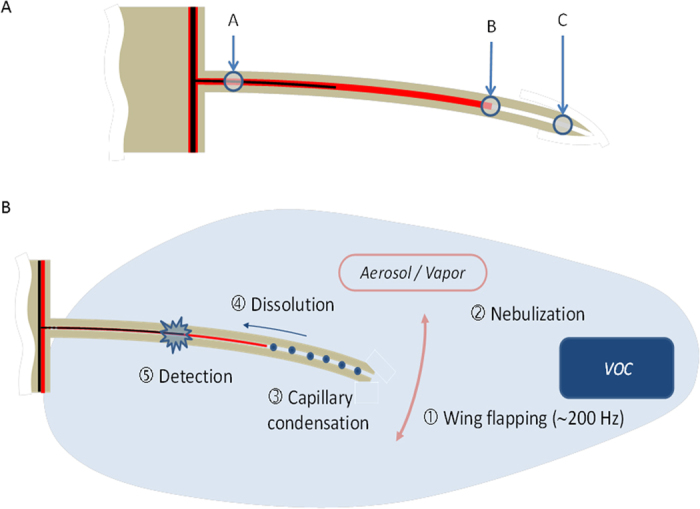
Schematic that summarizes the microphysics of the *Drosophila* wing chemosensory hair. (**A**) Schematic of a wing chemosensory hair structure showing a dendrite (black) surrounded by lymph (red). The top third of the hair from the base does not contain lymph and allows the exchange with the surrounding air environment. The sub-micronic diameter of this capillary structure exhibits the conditions for the physics of adsorption of volatile molecules. Letters represent the positions of the laser beam that generated the Raman spectra. (**B**) Schematic of the putative mechanism of a chemosensory hair showing an opened capillary structure. Inside the apical pore, the environmental volatile compounds are adsorbed, condensed and transferred by dissolution to the lymph (red) to access the dendrite (black). VOC denotes “volatile organic compounds”.

The insect respiratory system presents a complex network of tracheae (large tubes) and tracheoles (micro/nanometer diameter tubes) that irrigate the organs and the wing veins. To address whether the volatile molecules present in the air are condensed in tracheae/tracheoles, we tested the dynamics of benzaldehyde in the wing veins. The aldehyde domain of the molecule covalently binds to the amine groups of proteins. When attached to proteins, the benzyl domain autofluoresces with the excitation/fluorescence wavelength of FITC. In Fig. 5, these two chemical properties were used to analyse the concentration time course in the wing tracheal veins. We observed that the autofluorescence in living wings increased with the time of exposure to the environmental benzaldehyde (Fig. 5A). This experiment required that the flies be alive, as this phenomenon is abolished in dissected wings (Fig. 5B). Thus, an intact respiratory system of living animals was crucial for the condensation of chemical compounds in the wing trachea. Furthermore, the lymph that baths the dendrites in the thin hairs of the anterior wing margin is physically separated from the wing haemolymph circuit. Based on our Raman analysis that documents the probable existence of an air layer beneath the pore, we investigated whether this facilitates the diffusion/condensation of volatile molecules inside the hair. The thin hairs containing chemosensilla exhibited fluorescence after benzaldehyde exposure (Fig. 6A), unlike the stout bristles harbouring mechanosensilla (Fig. 6B). Again, fluorescence was present in the wing gustatory hairs of living animals but not in the dissected wings (Fig. 6C), suggesting that wing vibration and/or grooming contribute to the adsorption. More importantly, the fluorescence generated by benzaldehyde adsorption in the wing anterior margin vein correlated with the benzaldehyde concentration. With living animals, a strong fluorescence appeared in this vein proportional to the concentration of benzaldehyde, and this effect was abolished in cut wings (Fig. 6C,D). This strongly suggests that the trachea/tracheole networks concentrate/condense environmental volatile molecules in living animals. Our data also show that wing sensory hairs with a capillary-like structure should allow the concentration/condensation of many other environmental volatile molecules, including organic acids (acetic acid and propionic acid), amphoteric molecules (aldehyde compounds), or small molecules (CO_2_ or ethanol). This implies that the partially empty capillary structure of gustatory hairs likely influences wing chemoperception by modifying parameters such as local pH, local ionic forces and chemical concentrations. Figure 7 proposes a model inspired by the presented experiments. In summary, the tracheal system likely concentrates organic volatile compounds in the body, and this phenomenon is presently ignored by entomologists. The stimulation of wing sensilla neurons appears to be regulated by environmental molecules entering both through the pore of the gustatory hair and the trachea of the wing veins, creating a more complex system than previously thought.

## Discussion

The insect cardiac tube is a longitudinal dorsal tube running along the whole thorax to the extremity of the abdomen^34^,^35^,^36^,^37^,^38^. The abdominal region of the cardiac tube (the heart) contains a succession of small bulbs with two symmetric holes at their bases called *ostia*^34^,^35^,^36^,^37^,^38^. A circular flow of haemolymph is pushed from the abdomen to the head by the contraction of the heart tube, then returns to the abdominal organs and re-enters the dorsal heart tube through the *ostia*^39^. Furthermore, insects have several accessory hearts located at the bases of appendages such as the antennae and wings, perfusing those structures^39^,^40^,^41^,^42^,^43^,^44^. These pulsatile accessory hearts alleviate the efforts of the main abdominal heart and are autonomously regulated^34^,^35^,^36^,^37^,^38^,^39^. Consequently, the wing accessory hearts might have a stronger pulsatile rhythmicity when wings vibrate in flight compared to when they rest and might increase the concentration of volatile environmental molecules in the wing haemolymph via the tracheal system of pipes. This constitutes a sophisticated interphase between the respiratory and circulatory systems^36^,^42^,^45^ and suggests that environmental compounds access the wing gustatory receptors *via* the pores of the hairs and *via* the tracheal tubes embedded in the wing veins. Chemically, the wing is made of chitin, a polymer of N-acetylglucosamine^30^,^46^. The hairs are made of alkanes on the outer surface layer and a matrix of intertwined chitin chains on the inner layer^30^,^46^. The fact that the rigid structure of the hairs made of polymers of N-glucosamine and n-alkanes does not contain detectable proteins is an ideal system to probe the interior *via* Raman spectroscopy imaging with minimal interference. The Raman imaging technology successfully established the level of the air/lymph meniscus inside the hair. The Raman signals of water molecules were weak, which prompted us to focus on the strong signals of the amide bonds I and II and the aromatic amino acids tyrosine, tryptophan and phenylalanine between 1,200 and 2,000 cm^−1^. Although the green laser (514 nm) penetration was lower than the infrared laser (785 nm) penetration and the fluorescence was higher, both spectra are in accordance with each other. In parallel, a strong signal of carbon residues resulting from the breakdown of proteins under green laser irradiation was recorded. Altogether, the Raman experiments clearly indicated that the detected lymph was strongly dependent on the position of the laser beam along the bristle. We recorded high signals from the base up to half of the bristle and very low signals from two thirds along the bristle towards the apex. Considering the obtained Raman spectra of proteins and the drastic changes in signal intensity for both laser excitations, we can state that the hairs were partially filled with air. Rarely have electrophysiology studies been successfully performed in the wing tissue^12^. This strongly supports our conclusions for the wing nanoarchitecture of an air layer that exists beneath the pore of the hair, preventing the contact between dendrites and electrodes. In addition, we observed that autofluorescence generated by benzaldehyde was strong in the wing veins of living animals but was abolished in dissected wings. This indicates that the entire tracheal pipeline network is necessary to concentrate volatile molecules in wing veins in a dose-dependent manner, and this might rely on wing vibrations. These data also strongly support the argument that the physiology of gustatory sensilla in the wing is controlled by environmental molecules coming from the opened bristles and the trachea of the veins acting in synergy, both realizing the microphysics of porous materials and carbon nanotubes.

More importantly, we might question why evolution has selected gustatory cells in insect wings instead of olfactory ones. In mammals, the common paradigm is that volatile/ hydrophobic molecules activate olfactory receptors and hydrophilic molecules activate gustatory receptors. In fact, the boundaries between these two sensory modalities based on the physic-chemistry of stimuli do not apply in insects^47^. In insects, taste cells detect CO_2_ (in carbonate form)^48^, water (through osmosensitive ion channels)^49^, and pheromones^47^. The carboxylic acids such as acetic, propionic and citric acids amplify the bitter detection and inhibit the sweet perception^50^. All these molecules are volatile and convey taste cell signalling in insects. As for any porous material, the condensation of volatile molecules such as ethanol, water, and carboxylic acids in the wing tracheal system and the hollow wing taste hairs is likely a component of the process of the wing chemosensory detection. Vibrations of the wing creating vortexes and mixing molecules in the lymph/hemolymph are likely the other important component. To date, although the presence of taste sensilla in the *Drosophila* wing is fully accepted, we report here that these cells are functional and respond to sweet and bitter molecules as do proboscis taste cells. The wing taste receptors stimulated by hydrophilic tastants such as glucose and denatonium induce an intracellular calcium peak as they do in the proboscis. The access of tastants to wing receptors is likely provoked by leg grooming for *Drosophila* or by nebulization of microdroplets due to wing vibrations over flowers for insect pollinators. Altogether, these elements highlight the complex modality of taste in insects, which seems to result from the integration of coincidental signals triggered by volatile organic molecules, water vapour or CO_2_ along with classic hydrophilic tastants.

Our data suggest strongly that the nano architecture of the tracheal pipes and hollow hairs of the wing are intimately linked to the efficiency of chemical detection by wing sensilla. For this purpose, the emerging physics of carbon nanotubes and/or porous materials that highlight their impressive properties to concentrate or condense molecules present in the air^15^,^16^,^17^,^18^,^19^,^20^,^21^ can be used as a model to understand chemosensory physiology in insects. The functions of wing chemosensory neurons in insects and how they probe the complexity of a given chemical environment is currently unknown and understudied. We suggest that the *Drosophila* wing combines molecular condensation in submicron hollow tubes and trachea with wing vibrations acting as a vortex to solubilize molecules in the lymph/hemolymph. The presented data support the concept that wing sensilla receive chemical information from two channels: the pore of the hollow hair and the trachea pipes along the anterior wing margin at the base of the taste sensilla. In the future, we anticipate that this result could lead to a new generation of bio-inspired chemical sensors using high frequency bending nanotubes.

## Methods

### Drosophila strains and genetic constructs

G-CaMP is an engineered hybrid protein that is an efficient *in vivo* calcium sensor. Its gene combined with a yeast promoter (*UAS*) was introduced in *Drosophila*. This transgenic fly is publicly available from the Bloomington Drosophila Stock Center (stock n. 42037). G-CaMP is the fusion of calmodulin (CaM) and a modified GFP (N- and C-domains are fused to make an inverse and permuted protein^33^). In the absence of calcium release, this molecule emits a weak fluorescent signal. *GAL4* is an exogenous yeast transcription factor that recognizes the *UAS* motif and transcribes the downstream gene. For our experiments, we used the *Tubulin-GAL4* (*Tub-GAL4*) driver (Bloomington stock n. 5138) because no other driver, including the gustatory receptor drivers, produced strong enough expression in our calcium imaging procedure. Confocal microscopy observations were carried out in the dissected wings of *Tub-GAL4/* + *; UAS-G-CaMP/* +  flies ubiquitously expressing *UAS-G-CaMP*. The wings of five day old *Tub-GAL4/* + *; UAS-G-CaMP/* + flies were cut with a razor blade, mounted in water between a glass slide and a cover slip and observed with a confocal ZEISS LSM 510 META microscope (20× objective). A drop of the sugar/bitter solution (50 mM glucose and 1 mM denatonium in water) was deposited at the edge of the cover slip, and the time course of the fluorescence variation was recorded in real time.

### Raman spectroscopy analysis

A Labram HR800 Horiba Jobin-Yvon spectrophotometer was used with a 514 nm line of a tuneable air-cooled Melles-Griot Ar ion laser for excitation. The light intensity measured on the sample was maintained below 12 μW during the 30 s acquisition time. A near infrared laser diode at 785 nm was also used to avoid damaging the sample. The measured power at this wavelength was below 100 μW, with an acquisition time of 60 s. The analysis (excitation and collection) was performed for the excitation wavelength using a 100x objective with numerical aperture (NA) equal to 0.9. Under these conditions, the corresponding analysed area was below 1 μm^2^. Living *Drosophila* wings were cut with a razor at the wing/thoracic muscle junctions and immediately placed on a glass slide. The excitation Raman laser beam was focused on the bristles of the anterior wing margin. The Raman spectra are reported as the intensity of the scattered light on the *y*-axis and the Raman shift on the *x*-axis.

### Scanning electron microscopy imaging

SEM images were performed using an SU-70 Hitachi ultra-high resolution analytical field effect scanning electron microscope (FE-SEM) with a low operating voltage (5 kV), current of 49 mA and chamber pressure of 7 × 10^−4^ Pa. *Drosophila* wings were cut, fixed on an aluminium holder with carbon tape and metalized with a 20-nm osmium coating by sputtering techniques.

### Benzaldehyde exposure and wing autofluorescence

The flies (25, males and females, five days old) were placed in *Drosophila* vials (300 ml volume) in which 1 to 50 μl of benzaldehyde were deposed on a piece of paper soaked in water. The wings were cut according to the times indicated in the figure legends, and the fluorescence corresponding to the spectral properties of FITC (fluorescein isothiocyanate, excitation at 490 and emission at 520 nm) was analysed using the Zeiss Axiocam MRm ApoTome.2 fluorescence microscope. These experiments were designed based on the model of porous material in which the atmospheric gas condenses in the liquid phase inside the capillaries according the physical laws as described by the Kelvin equation: ln Pv/Psat = −(2HγVl)/RT where: Pv = equilibrium vapour pressure; Psat = saturation vapour pressure; H = mean curvature of meniscus; γ = liquid/vapour surface tension; Vl = liquid molar volume; R = ideal gas constant; T = temperature^22^,^23^,^25^.

## Additional Information

**How to cite this article**: Valmalette, J. C. *et al.* Nano-architecture of gustatory chemosensory bristles and trachea in *Drosophila* wings. *Sci. Rep.* **5**, 14198; doi: 10.1038/srep14198 (2015).
